# SARS-CoV-2 seroepidemiology in paediatric population during Delta and Omicron predominance

**DOI:** 10.1017/S0950268822001601

**Published:** 2022-10-19

**Authors:** Filippos Filippatos, Elizabeth-Barbara Tatsi, Charilaos Dellis, Dimitra-Maria Koukou, Christos Papagiannopoulos, Alexandra Margeli, Tania Siahanidou, Christina Kanaka-Gantenbein, Vasiliki Syriopoulou, Athanasios Michos

**Affiliations:** 1 First Department of Pediatrics, Infectious Diseases and Chemotherapy Research Laboratory, Medical School, National and Kapodistrian University of Athens, “Aghia Sophia” Children's Hospital, Athens, Greece; 2 University Research Institute of Maternal and Child Health and Precision Medicine, Athens, Greece; 3 First Department of Pediatrics, Medical School, National and Kapodistrian University of Athens, “Aghia Sophia” Children's Hospital, Athens, 11527, Greece; 4 Department of Clinical Biochemistry, “Aghia Sophia” Children's Hospital, Athens, Greece

**Keywords:** Children, COVID-19, Omicron variant, SARS-CoV-2, seroepidemiology

## Abstract

Limited prospective severe acute respiratory syndrome coronavirus 2 (SARS-CoV-2) data in children regarding the impact of Omicron variant in seropositivity have been reported. We investigated SARS-CoV-2 seropositivity in children between 1 September 2021 and 30 April 2022, representing Delta and Omicron predominance periods. Serum samples from children admitted to the major tertiary Greek paediatric hospital for any cause, except for COVID-19, were randomly collected and tested for SARS-CoV-2 natural infection antibodies against nucleocapsid antigen (Elecsys® Anti-SARS-CoV-2 reagent). A total of 506/1312 (38.6%) seropositive children (0–16 years) were detected (males: 261/506(51.6%); median age (IQR): 95.2 months(24–144)). Seropositivity rates (%) increased from Delta to Omicron period from 29.7% to 48.5% (*P*-value<0.0001). Seropositivity increased for all age groups, except for the age group of 0–1 year (*P*-value:0.914). The highest seropositivity rate was detected in April 2022 (52.6%) and reached 73.9% specifically for the age group 12–16 years. No significant differences were detected in seropositivity with respect to gender, origin, or hospitalisation status. Median (IQR) antibody titres were higher in the Omicron *vs.* Delta period in all age groups, especially in 12–16 years [32.2 COI (7–77.1) *vs.* 11.4 COI(2.8–50.2), *P*-value:0.009). During Omicron variant period increased SARS-CoV-2 seropositivity was detected in paediatric population, especially in adolescents, implicating either increased transmissibility or reinfection rates.

## Introduction

Severe acute respiratory syndrome coronavirus 2 (SARS-CoV-2) rapidly evolves, gathering significant mutations and leading to new variants of concern [1]. The predominant SARS-CoV-2 variants of concern are Delta (B.1.617.2 lineage) and Omicron (B.1.1.529 lineage). Delta variant was first identified in December 2020 and became the most prevalent variant worldwide until the emergence of the Omicron variant in December 2021 [2]. Omicron variant predominance resulted in a robust increase in reported infections and reinfections in all age groups, including children [3].

According to the American Academy of Pediatrics, by June 2022 there are more than 13 million laboratory confirmed coronavirus disease 2019 (COVID-19) cases in children, representing approximately 20% of total COVID-19 cases [3]. After Delta emergence, the proportion of paediatric COVID-19 tests has increased as conditions for testing were extended [4]. The reported case surveillance data in children, based on molecular or antigen testing, underestimate the overall burden of COVID-19, as only a small proportion of acute infections are symptomatic, diagnosed and reported [5].

Seroepidemiological studies are an indispensable tool for tracking the evolution of the COVID-19 pandemic in children and assist in interpreting the true extent of the pandemic in the paediatric population [6]. In a large-scale study from the United States of America (USA), the average seroprevalence rates after natural infection were found to be superior in children <18 years of age compared to adults 18–49, 50–64 and over 65 years (75% *vs* 64% *vs* 50% *vs* 33%, respectively) [7]. In a previous study from our group, SARS-CoV-2 seropositivity between May 2020 and August 2021 increased from 4% to 17.2%, respectively [8].

The purpose of the study was to prospectively study evolution of SARS-CoV-2 seropositivity in children 0–16 years of age during the Delta and Omicron variant period and to investigate the role of different epidemiological parameters.

## Materials and methods

### Study Design and participants

This was a prospective cohort study including children who presented to the emergency department or were hospitalised to ‘Aghia Sophia’ Children's Hospital for any reason, except COVID-19. This is a 750-bed tertiary paediatric hospital, which is the largest hospital for children in Greece and a COVID-19 reference centre for the paediatric population of Athens, Greece.

To evaluate the seropositivity (%) of children after natural infection, serum samples were prospectively collected from 01/09/2021 to 30/04/2022. Each month, approximately 150–180 serum samples were randomly collected from the Department of Clinical Biochemistry. Serum samples were residual sera that were ordered from paediatricians for any medical reason from hospitalised children or children from the emergency department. Epidemiological parameters, which included age, gender, origin and hospitalisation status, were recorded. Further laboratory testing of serum samples was performed anonymously using an identification code.

Children with proven SARS-CoV-2 infection either with SARS-CoV-2 molecular testing or with the rapid test were excluded from the study. If a child was admitted to the hospital more than once within the study period and the results of his antibody test results were positive, only those of the first positive result were included in the analysis. Serum samples from children admitted to the Departments of Oncology, Bleeding Disorders or B-Thalassemia were excluded from the study due to transfusions or immunocompromised conditions that may affect antibody detection.

The study period was divided into two 4-month different subperiods, representing the predominance of two different SARS-CoV-2 variants of concern, Delta and Omicron, in Greece: 01/09/2021-31/12/2021 (Delta predominance) and 01/01/2022-30/04/2022 (Omicron predominance). Children from different age groups were enrolled in this study and were categorised as infants (0–1 year), toddlers (1–4 years), pre-school (4–6 years), primary school (6–12 years) and adolescents (12–16 years).

### Ethics Approval

The study protocol was in accordance with the Declaration of Helsinki of 1964 and its subsequent amendments or comparable ethical standards and was approved by the Scientific and Bioethics Committee of the ‘Aghia Sophia’ Children's Hospital (No. 25609).

### Antibody Detection assay

The detection of SARS-CoV-2 antibodies was performed in the Infectious Diseases Laboratory, First Department of Pediatrics, Medical School of the National and Kapodistrian University of Athens, Serum samples were tested using Elecsys® Anti-SARS-CoV-2 (Roche Diagnostics, Basel, Switzerland) reagent on a Cobas e 411 immunoassay analyser for the semi-quantitative detection of total antibodies (IgA, IgM and IgG) against SARS-CoV-2 nucleocapsid protein according to the manufacturer's instructions.

### Statistical Analysis

The data are presented as percentage (%), median, and interquartile range (IQR) because of a skewed distribution. Normality was tested with Anderson–Darling and Shapiro–Wilk tests. The comparison of continues data was tested with the nonparametric test Wilcoxon rank sum test and Kruskal–Wallis test. The multiple comparison was tested with pairwise Wilcoxon rank sum test with Bonferroni correction. The categorical data were tested with the *X*^2^ test, Fischer's exact test and the *X*^2^ goodness of fit test. Statistical analysis was performed using R version 4.1.2 and the significance level was set at a *P-*value of <0.05.

## Results

### Study Population

From 1 September 2021 to 30 April 2022, serum samples from 1312 children were tested; 693/1312 (52.8%) in Delta and 619/1312 (47.2%) in the Omicron period, respectively. The epidemiological characteristics of the study population in each study period are presented in Table 1. Among the 1312 children, 702 (53.5%) were males, and the median age (IQR) of the study population was 84 months (22.8–144). More specifically 251(19.1%) were 0–1-year-old, 247 (18.8%) were 1–4 years old, 132 (10.1%) were 4–6 years old, 382 (29.1%) were 6–12 years old, 300 (22.9%) were 12–16 years old. Regarding hospitalization status and origin, 914 (69.7%) children were hospitalised and 1000 (76.2%) were Greek. The median age (IQR) of the children in the Delta and Omicron periods was 84 months (20.8–144.6) and 84 months (24–144), respectively (*P*-value: 0.72).

**Table 1.** Distribution of epidemiological characteristics of the study population in September 2021–December 2021 (Delta period) and January 2022–April 2022 (Omicron period)

Variable	Categories		Study period		
		Total	Delta period	Omicron period	*P*-value
		*n*(%)	*n*(%)	*n*(%)	
Children tested		1312	693	619	
Median age (IQR)		84 (22.8–144)	84 (20.8–144)	84 (24–144)	0.720
Seropositive		506/1312 (38.6)	206/693 (29.7)	300/619 (48.5)	**<0**.**0001**
Gender	Female	245/610 (40.2)	101/308 (32.8)	144/302 (47.7)	**<0**.**001**
	Male	261/702 (37.2)	105/385 (27.3)	156/317 (49.2)	**<0**.**001**
	*P*-value	0.293	0.135	0.764	
Age group (years)	0–1 years	80/251 (31.9)	45/144 (31.3)	35/107 (32.7)	0.914
	1–4 years	91/247 (36.8)	37/123 (30.1)	54/124 (43.6)	**0**.**039**
	4–6 years	52/132 (39.4)	16/65 (24.6)	36/67 (53.7)	**0**.**001**
	6–12 years	159/382 (41.6)	55/198 (27.8)	104/184 (56.5)	**<0**.**001**
	12–16 years	124/300 (41.3)	53/163 (32.5)	71/137 (51.8)	**0**.**001**
	*P*-value	0.109	0.746	**0.001**	
Department	Hospitalised	351/914 (38.4)	160/528 (30.3)	191/386 (49.5)	**<0**.**001**
	Non-hospitalised	155/398 (38.9)	46/165 (27.9)	109/233 (46.8)	**<0**.**001**
	*P*-value	0.902	0.619	0.570	
Origin	Greek	377/1000 (37.7)	161/550 (29.3)	216/450 (48)	**<0**.**001**
	non-Greek	129/312 (41.4)	45/143 (31.5)	84/169 (49.7)	**0**.**002**
	*P*-value	0.276	0.682	0.774	

IQR: interquartile range.

The categorical data were tested with the *X*^2^ test. The comparison of median age was tested with Wilcoxon rank sum test. Statistically significant (*P*-value <0.05) differences are marked in bold.

A total of 506/1312 (38.6%) seropositive children were detected with median age (IQR) of 95.2 months (24–144). The median age (IQR) of seropositive children was 84 months (18–145.2) in Delta and 96 months (40.6–144) in the Omicron period, respectively (*P*-value: 0.174). Among the 506 children, 261/506 (51.6%) were males, 351/506 (69.4%) were hospitalised and 377/506 (74.5%) were of Greek origin (Table 1).

### Seropositivity In different age groups per subperiod and per month

**Fig. 1.**
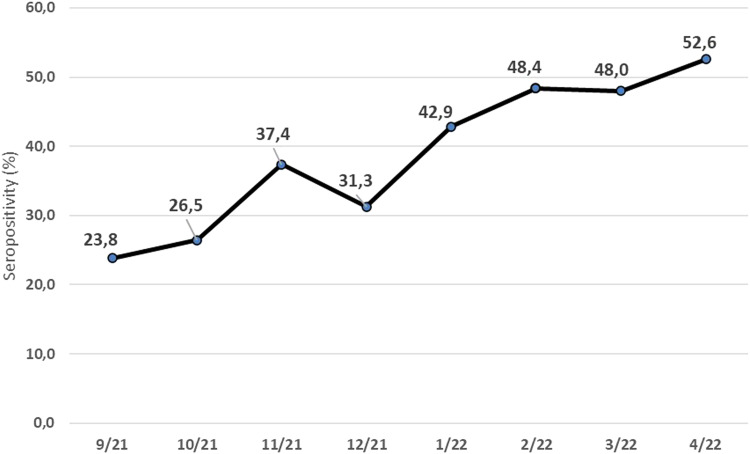
Seropositivity (%) per month in 1312 children from September 2021 to April 2022 in Athens metropolitan area.

SARS-CoV-2 seropositivity significantly increased from 29.7% (206/693) in Delta to 48.5% (300/619) in Omicron period for the whole study population (*P*-value < 0.0001) (Table 1). SARS-CoV-2 seropositivity during each study month also varied significantly regardless of age (*P*-value<0.0001) and is presented in Figure 1. The lowest seropositivity was detected in September 2021 (23.8%, 45/189) and the highest was detected in April 2022 (52.6%,82/156) (Fig. 1).

SARS-CoV-2 seropositivity detected in Delta and Omicron periods per age group are presented in Table 1. Regarding seropositivity rates per subperiod, no significant differences were detected in Delta period (*P*-value: 0.746) among the different age groups, but significant differences were detected in Omicron period (*P*-value: 0.001). Compared to the period 1 May 2021–31 August 2021 (our previously published data [8]), seropositivity rates increased for all age groups. The seropositivity of SARS-CoV-2 gradually increased from Delta to the Omicron period for all age groups, except for the age group of 0–1 years (*P*-value: 0.914, Table 1). In Delta period, the age group 12–16 years had the highest seropositivity rate (32.5%, 53/163), followed by the 1–4 years (30.1%, 37/123). In Omicron period, the age group 6–12 years had the highest seropositivity rate (56.5%,104/184), followed by the 4–6 years (53.7%, 36/67).

**Fig. 2.**
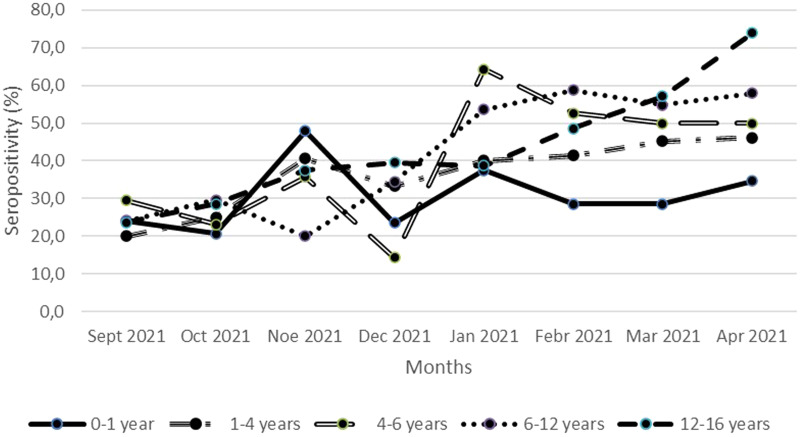
Seropositivity per month per age group from September 2021 to April 2022 in Athens metropolitan area.

SARS-CoV-2 seropositivity per age group varied significantly (*P*-value: 0.017) for each month of the study period (Fig. 2). Regarding seropositivity per month, significant differences were detected in the age groups 4–6 years (*P*-value: 0.045), 6–12 years (*P*-value<0.001) and 12–16 years (*P*-value: 0.002). The highest seropositivity rate was detected in April 2022 in the age group 12–16 years (73.9%, 17/23) and the lowest seropositivity rate was detected in December 2021 in the age group 4–6 years (14.3%, 3/11) (Fig. 2). The highest seropositivity rates per month for each age group were in November 2021 for the year 0–1 (47.9%, 23/48), in January 2022 for the 4–6 years (64.3%, 9/14) and in April 2022 for the 1–4 (46.2%, 18/39), 6–12 (58%, 29/50) and 12–16 years (73.9%, 17/23) (Fig. 2). The lowest seropositivity rates per month for each age group were in October 2021 for the 0–1 year (20.7%, 6/29), in September 2021 for the 1–4 (20%, 6/30) and 12–16 years (23.7, 9/38), in December 2021 for 4–6 years (14.3%, 3/21) and in November 2021 for 6–12 years (20%, 7/35) (Fig. 2).

### Seropositivity Per gender, origin, hospitalisation status and paediatric departments

No significant differences were detected between seropositive males and females (*P*-value: 0.293), seropositive Greek and non-Greek (*P*-value 0.276) and seropositive hospitalised and non-hospitalised children (*P*-value: 0.902) during the whole study period (Table 1). Seropositivity was significantly higher in Omicron compared to the Delta period regardless of gender (*P*-value<0.001), origin (*P*-value<0.002) or hospitalisation status (*P*-value<0.001) (Table 1).

SARS-CoV-2 seropositivity did not significantly vary among the different paediatric departments of the hospital for the entire study period (*P*-value: 0.267) or for each subperiod (*P*-values: 0.072 for Delta and 0.455 for Omicron period, respectively). More specifically, seropositivity throughout the study period was 40.7% (227/681) in general paediatric departments, 37.8% (14/37) in paediatric intensive care unit (PICU), 32.1% (18/56) in neonatal intensive care unit (NICU), 27.6% (27/98) in surgical departments (including cardiothoracic surgery, neurosurgery, orthopaedics, urology, ophthalmology, plastic surgery and otorhinolaryngology departments), 35.7% (15/42) in the cardiology and neurology departments.

SARS-CoV-2 seropositivity increased significantly from Delta to Omicron period in general paediatric departments (33% (129/391) *vs.* 51% (148/290), *P*-value<0.001), in surgical departments (17.3% (9/52) *vs.* 39.1% (18/46), *P*-value: 0.029) and in cardiology and neurology departments (10% (2/20) *vs.* 59.1% (13/22), *P*-value: 0.001). No significant differences in seropositivity were detected in PICU (28.6% (6/21) *vs.* 50% (8/16), *P*-value: 0.323) and NICU between the Delta and Omicron period (31.85 (14/44) *vs.* 33.3% (4/12), *P*-value> 0.999).

### Antibody Titres per age group

**Fig. 3.**
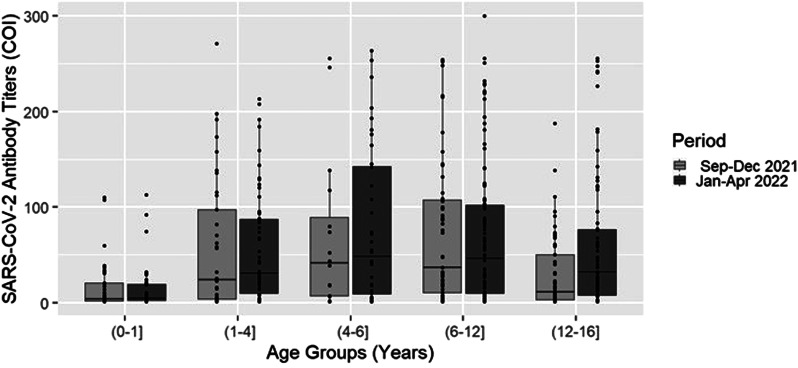
Median antibody titres per age group in September 2021–December 2021 (Delta period) and January 2022–April 2022 (Omicron period) in Athens Metropolitan area. Lines represent median antibody titre values and bars represent interquartile range (IQR) values (COI, cut-off index).

Antibody titres per age group for each study period are presented in Figure 3. The median Abs titre per age group for both subperiods was 4.3 COI (2.1–20.4) in the 0–1 year, 28.1 COI (4.3–90.4) in the 1–4 years, 43.6 COI (7.2–126.4) in 4 to 6 years, 44.1 COI (10.7–103.1) in 6 to 12 years and 22.4 COI (4.9–62.2) in 12 to 16 years (*P*-value<0.0001).

Antibody titres per age group significantly varied in both study subperiods (*P*-value<0.0001). Compared to the Delta period, median antibody titres were significantly higher in the Omicron period in 12–16 years (11.4 COI (2.8–50.2) *vs.* 32.2 COI (7–77.1), *P*-value: 0.009) (Fig. 3). No significant differences were detected between the two study periods regarding median (IQR) antibody titres in the other age groups (*P*-value>0.497) (Fig. 3). In Omicron period, the lowest and highest median antibody titres were observed in 0–1 year (4.5 COI (2.5–19.6)) and 4–6 years (48.4 COI (9.1–142.8)) (*P*-value<0.0001), respectively.

## Discussion

In the present study, we prospectively evaluated the seropositivity of SARS-CoV-2 in a large paediatric population to assess the impact of Omicron compared to Delta variant on the natural infection of paediatric SARS-CoV-2 and investigate possible associations with epidemiological characteristics. The prospective collection of routine residual serum samples from the Biochemistry department is a practical method for the estimation of SARS-CoV-2 serosurveillance in children as it does not burden paediatric patients with any extra blood sampling and was also used not only in our previously published study [8] but also in other studies as well [9].

The present study showed a significant increase in seropositivity rates in paediatric population from the Delta to Omicron period. By April 2022, more than half of the children included in the study had serological evidence of previous exposure to SARS-CoV-2. These findings indicate the high infection rate of the Omicron variant in children and are in line with other contemporary seroprevalence surveys from the USA and Europe [10, 11]. In the USA, approximately 75% of children and adolescents had detectable antibodies for natural SARS-CoV-2 infection by February 2022, with a marked increase in seropositivity being detected after the appearance of the Omicron in December 2021 [10]. At the beginning of 2022 in Finland, seropositivity in children and young adults <30 years increased considerably to 40% and was even higher compared to adults 30–45 years (30%) and >45 years (20%) [11].

The release of restriction measures, the opening alongside the difficulty of young children to comply with social distancing and indoor mask-wearing recommendations can at least partially justify this finding. Compared to Delta, Omicron is characterised by a higher replication rate, increased transmissibility, and significant vaccine-induced immune escape strategies alongside other variants of concern [1, 12, 13].

In April, most of the adolescents had detectable antibodies attributed to SARS-CoV-2 natural infection, representing the highest seropositivity rate during the 8-month study period (73.9%). According to WHO and other studies, adolescents are more likely to be seropositive than younger children [10, 14]. Furthermore, SARS-CoV-2 viral load and transmissibility of adolescents is comparable or even higher to those in adults [15].

Neonates and infants did not only have the lowest seropositivity rates per month and per study subperiod, but also mounted the lowest median antibody titres both during Delta and Omicron concurrence. Similar results were also observed in our previously seroprevalence study between 1 May 2021–31 August 2021 during the other variants' predominance. For this age group, a randomly detected antibody titre cannot allow us to determine whether there was a previous SARS-CoV-2 infection in the child or in the mother during pregnancy. Maternal IgG levels usually peak at least 30 days after the onset of symptoms, but the highest transfer is achieved when the onset of the infection is at least 60 days before labour [16, 17]. More studies should investigate the role of passive newborn immunity in the protection of SARS-CoV-2 infection for Omicron or other future variants, as factors affecting transplacental transfer have not yet been clearly elucidated.

With the exception of neonates and infants, seropositive children ≥1 year old, and especially adolescents 12–16 years, mounted higher median antibody titres in the Omicron compared to Delta period. This could be a reflection of the higher secondary attack and reinfection rate of Omicron in households compared to previously circulating variants, which has been highlighted by other large-scale studies [18, 19]. Even before Omicron, a prospective surveillance study from England demonstrated that the rate of children's reinfection was relatively low but related to community infection rates and positively associated with age [20]. Children previously infected by variants other than Omicron retain antibody levels and antigen-specific CD4^+^ T-cells for at least 12 months after the initial infection, but the decrease of antibody neutralising activity and CD8^+^ cytotoxic effects make them prone to reinfections from variants that have immune-mediated evasion strategies, such as Omicron [21].

Another possible reason that could justify the difference between the median antibody titres of the Delta and Omicron period in our study is that their detection was close to seroconversion or the peak of antibody levels for children infected with Omicron. In the USA, approximately one-third of seroconversions in children and adolescents occurred between December 2021 and February 2022, which was during Omicron predominance, and resulted in seropositivity rates of 75% [10].

In this study, no significant differences were detected in seropositivity rates with respect to gender, origin or hospitalisation status. COVID-19 seropositivity does not differ between paediatric males and females from the beginning of the pandemic in Greece [8] and this finding is also consistent with laboratory-confirmed SARS-CoV-2 cases in Greece (based on Greek National Public Health Organization (GNPHO) [22]) and other countries' surveillance systems or seroprevalence studies [7, 23]. By August 2021, non-Greek and hospitalised children were more likely to be seropositive in Greece [8], however the noteworthy increase of total paediatric cases in Omicron period seems to have eliminated those differences. Probably a larger number of patients with these characteristics could more accurately determine whether origin and hospitalisation play a key role in SARS-CoV-2 seropositivity after natural infection.

The study has several limitations, most of which are precluded in the majority of published seroprevalence studies [6]. The incommensurate distribution of the study population, mainly regarding origin, hospitalisation status and hospital departments, did not allow us to detect other possible important differences or associations. Therefore, this is the largest prospective seroepidemiological study in the paediatric population of Greece in terms of study population and duration. It is also one of the few studies with prospective design of Omicron in children worldwide. The results of our study may offer valuable information on the impact of Omicron in paediatric population of Athens but should be interpreted with caution since they are not representative of other areas of Greece.

## Conclusions

The onset of the Omicron variant was associated with a sharp increase in reported SARS-CoV-2 infection rates in many countries. Worldwide data are supportive of increased transmissibility, immune evasion strategies, reinfection potential and replication advantages of Omicron over the Delta and other variants, but evidence regarding the impact of Omicron in children is limited. This seroepidemiological study shows that in Omicron period, SARS-CoV-2 seropositivity in paediatric population considerably increased, notably among adolescents, suggesting either enhanced transmissibility or reinfection rates. Since most children remain asymptomatic or mildly symptomatic during the acute phase of COVID-19, continuous surveillance of seroepidemiological data in children is required to determine the true transmission of SARS-CoV-2 in paediatric population.

## Data Availability

The data that support the findings of this study are available from the corresponding author upon reasonable request.
